# Resveratrol alleviates FFA and CCl_4_ induced apoptosis in HepG2 cells via restoring endoplasmic reticulum stress

**DOI:** 10.18632/oncotarget.16460

**Published:** 2017-03-22

**Authors:** Fenghua Li, Yang Yang, Lili Yang, Kai Wang, Xing Zhang, Yunlong Zong, Yixin Ding, Changhong Wang, Li Zhang, Guang Ji

**Affiliations:** ^1^ Experiment Center for Science and Technology, Shanghai University of Traditional Chinese Medicine, Shanghai, China; ^2^ Institute of Digestive Diseases, China-Canada Center of Research for Digestive Diseases, Shanghai University of Traditional Chinese Medicine, Shanghai, China; ^3^ Institute of Chinese Materia Medica, Shanghai University of Traditional Chinese Medicine, Shanghai, China

**Keywords:** apoptosis, endoplasmic reticulum stress, resveratrol, inflammation, cytokines

## Abstract

Cell apoptosis often induces inflammation and injury in the liver, with endoplasmic reticulum (ER) stress as the most possible reason. Resveratrol (RSV) has been shown to prevent hepatic steatosis and alleviate apoptosis, however, the exact mechanisms underlying the effects still need to be explored. Here we co-cultured HepG2 cells with free fatty acid (FFA) solution (oleic acid: palmitic acid = 2:1) and then exposed to a carbon tetrachloride (CCl4) solution to induce apoptosis. To evaluate the therapeutic effects, RSV (2.5 μM, 5 μM, 10 μM) was added to the cells. Results showed that HepG2 cells co-cultured with FFA exhibited lipid infiltration and were susceptible to apoptosis upon exposure to the CCl4 solution. The expression of molecules related to apoptosis (Caspases, Bcl-2/Bax) and ER stress (GRP78, IRE1, ATF6, PERK, et al.) was all significantly decreased upon RSV treatment. We further inhibited GRP78 by siRNA, results showed that the anti-apoptotic effect of RSV still maintained under GRP78 siRNA condition. Our data demonstrated that lipid accumulated HepG2 cells were susceptible to injury, and RSV could improve apoptosis in FFA and CCl4 stressed cells, which partially via restoring ER function.

## INTRODUCTION

Liver is the primary organ of lipid metabolism and plays a major role in detoxification [1]. Hepatocytes are the major functional cells in the liver and compose 70-85% of the parenchymal tissue. Metabolic disruption in the liver can be caused by multiple factors, such as obesity, diabetes, and viral infection [2]. Nutrient stressed hepatocytes are easily to be steatosis, with non-alcoholic fatty liver disease (NAFLD) as the representative liver diseases that associated with steatosis. NAFLD is defined as fat accumulation exceeding 5% of the whole weight of the liver, and it significantly increases the risk of chronic hepatic diseases in patients [3].

Steatosis is considered to diminish the function of hepatocytes in metabolizing, detoxifying, and inactivating exogenous compounds, rendering the liver to be more sensitive to toxin-induced damage [4]. Therefore, NAFLD can progress from simple steatosis to non-alcoholic steatohepatitis (NASH), cirrhosis, and even hepatocarcinoma. Lipid accumulated hepatocytes are prone to apoptosis, which is one of the most prominent features in the development and progression of liver diseases. Various types of cell stress can cause hepatocyte apoptosis, but the exact mechanisms have yet to be determined. Recently, endoplasmic reticulum (ER) stress has been reported to be one of the most likely causes [5]. On the one hand, ER is a vital organelle that responsible for protein, lipid, and steroid synthesis and metabolism, making ER especially sensitive to the metabolic disorders associated with NAFLD. On the other hand, the ER is responsible for the regulation of calcium homeostasis, post-translational modifications and the folding of newly synthesized proteins, all of which are related to cell survival [6–8]. Therefore, ER stress is more likely to cause hepatocyte apoptosis in patients with NAFLD that was induced by risk factors.

Resveratrol (3,5,4’-trihydroxystilbene, RSV) is a natural compound with a polyphenolic structure, it is found in grapes and red wine and has been indicated to obtain a wide range of biological effects, such as lifespan extension [9, 10], cardioprotective [11, 12], anticancer [13], anti-inflammatory [14], and antioxidant [15] properties. In addition, RSV was reported to improve NAFLD in animals, possibly via regulating metabolic homeostasis, improving inflammation and oxidative stress [16–20]. However, the regulatory effect of RSV on ER stress is relatively complex. Cancer research has indicated that RSV plays a role in inducing ER stress to promote cancer cell apoptosis [21–23]. Other research has shown that RSV improves cardiomyocyte hypertrophy and inflammation via inhibiting ER stress [24–26]. In rats fed a high-fat diet, RSV could prevent hepatic steatosis and ER stress [27], but the mechanisms require further study.

Considering the potential role of ER stress on apoptosis and the importance of hepatocytes in liver injury, we specifically studied the role of RSV on apoptosis in hepatocytes. By evaluating the effects and inhibiting ER chaperon protein, we looked insight into the mechanisms underlying the anti-apoptotic capacity of RSV.

## RESULTS

### Lipid-stressed cells were more susceptible to apoptosis

**Figure 1:**
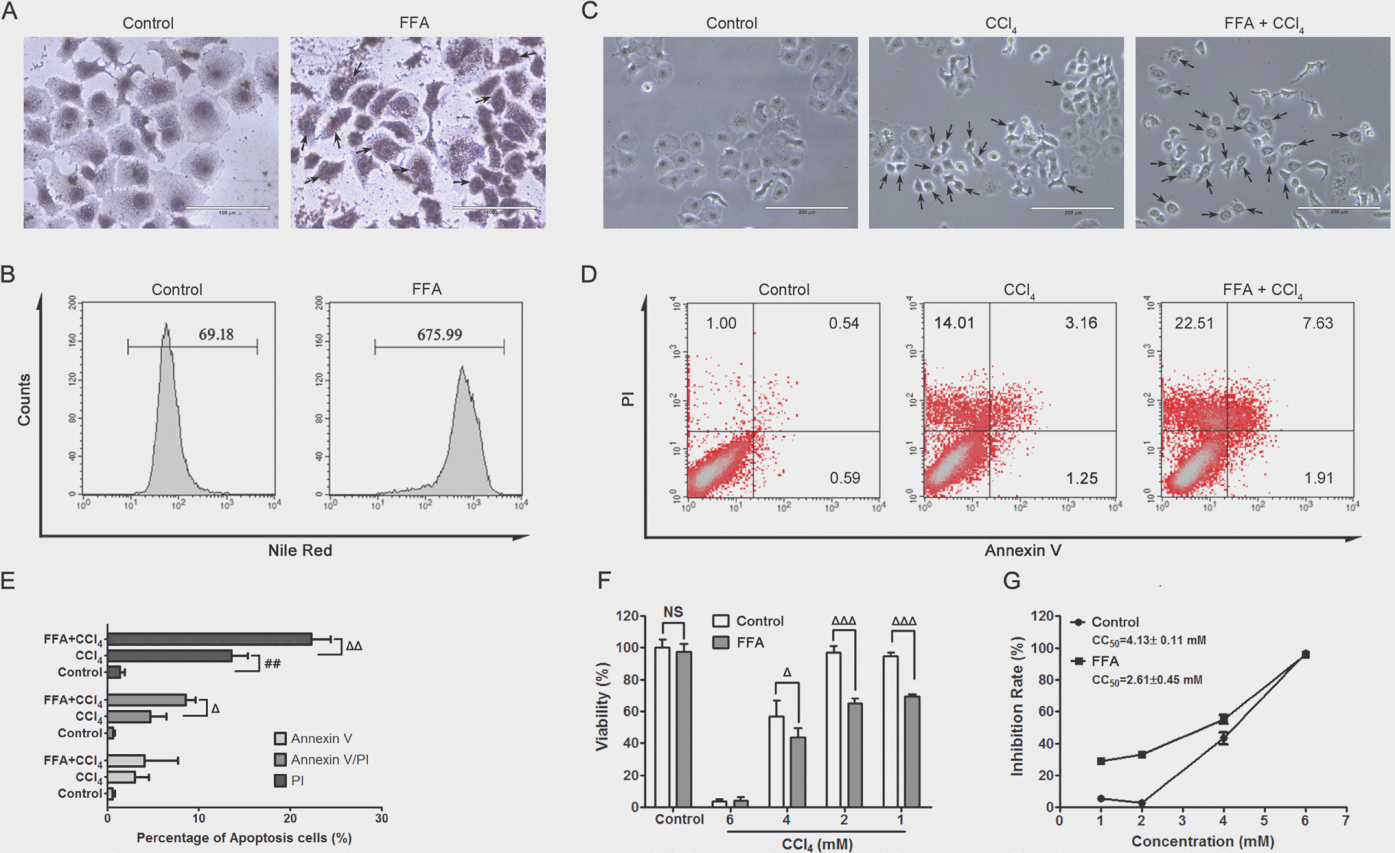
FFA and CCl_4_ inudce cell apoptosis The effects of FFA treatment on co-cultured HepG2 cells with 0.5 mM oleic acid and 0.25 mM palmitic acid for 24 h, and lipid accumulation was determined by Oil red O (**A**) and Nile red staining (**B**). (HepG2 cells were treated with CCl_4_ (4 mM)) alone for 24 h, or 24 h FFA incubation followed by another 24 h CCl_4_ (4 mM) co-culture Representative images of the morphological change were obtained (**C**), the arrows indicate the typical apoptotic cells, magnification, 200×. Cell apoptosis was analysed by flow cytometry using the annexin V (FITC)/PI binding assay (**D**), and the percentage of apoptotic cells was calculated (**E**). The effects of on cell viability (**F**) upon CCl_4_ treatment (1 mM, 2 mM, 4 mM and 6 mM for 24 h) was determined by the crystal violet colorimetric assay, and was calculated CC_50_ (**G**). The data were presented as the mean ± sd of three independent experiments. Statistical analysis was performed by comparing the viability of cells treated with or without FFA. ^##^*p* < 0.01, *vs* control cells; ^∆^*p* < 0.05, ^∆^^∆^*p* < 0.01, ^∆^^∆^^∆^*p* < 0.001 *vs* CCl_4_ stimulated cells; NS = no significant difference.

HepG2 cells co-cultured with free fatty acid (FFA) solution for 24h showed obvious lipid droplet accumulation, as identified by Oil Red O staining (Figure 1A). By staining with Nile red, lipid content in the cells was quantified by flow cytometry. Results indicated that the mean fluorescence intensity (MFI) was significantly increased in the FFA co-cultured cells compared to that in control cells (Figure 1B). Followed by co-culturing with FFA solution, the cells were further exposed to carbon tetrachloride (CCl_4_) solution to induce apoptosis, and we also set cells with CCl_4_ stimulation alone. Microscopy observations indicated that both CCl_4_ and FFA+CCl_4_ stimulated cells failed to adhere completely and showed considerable shrinkage. Typical morphological characteristics of apoptosis (nuclear fragmentation and chromosome condensation) were found in the FFA+CCl_4_ cells (Figure 1C). Annexin V/PI staining was used to quantitatively analyse apoptosis, and the results showed that the proportion of both early and late apoptotic cells dramatically increased in FFA+CCl_4_ cells in comparison with the CCl_4_ stimulation cells (Figure 1D and 1E).

We have also detected the cell viability, compared with control cells, FFA alone or low concentration CCl_4_ (1 mM, 2 mM) alone did not obviously affect cell viability in HepG2 cells, however, the same dosage of CCl_4_ caused significantly cell viability decrease in FFA stressed cells (Figure 1F). The CC_50_ value of CCl_4_ decreased from 4.13 mM in control cells to 2.61 mM in FFA stressed cells, indicating that the steatotic HepG2 cells were more susceptible to cell injury (Figure 1F and 1G).

### Cell apoptosis was associated with ER stress

**Figure 2:**
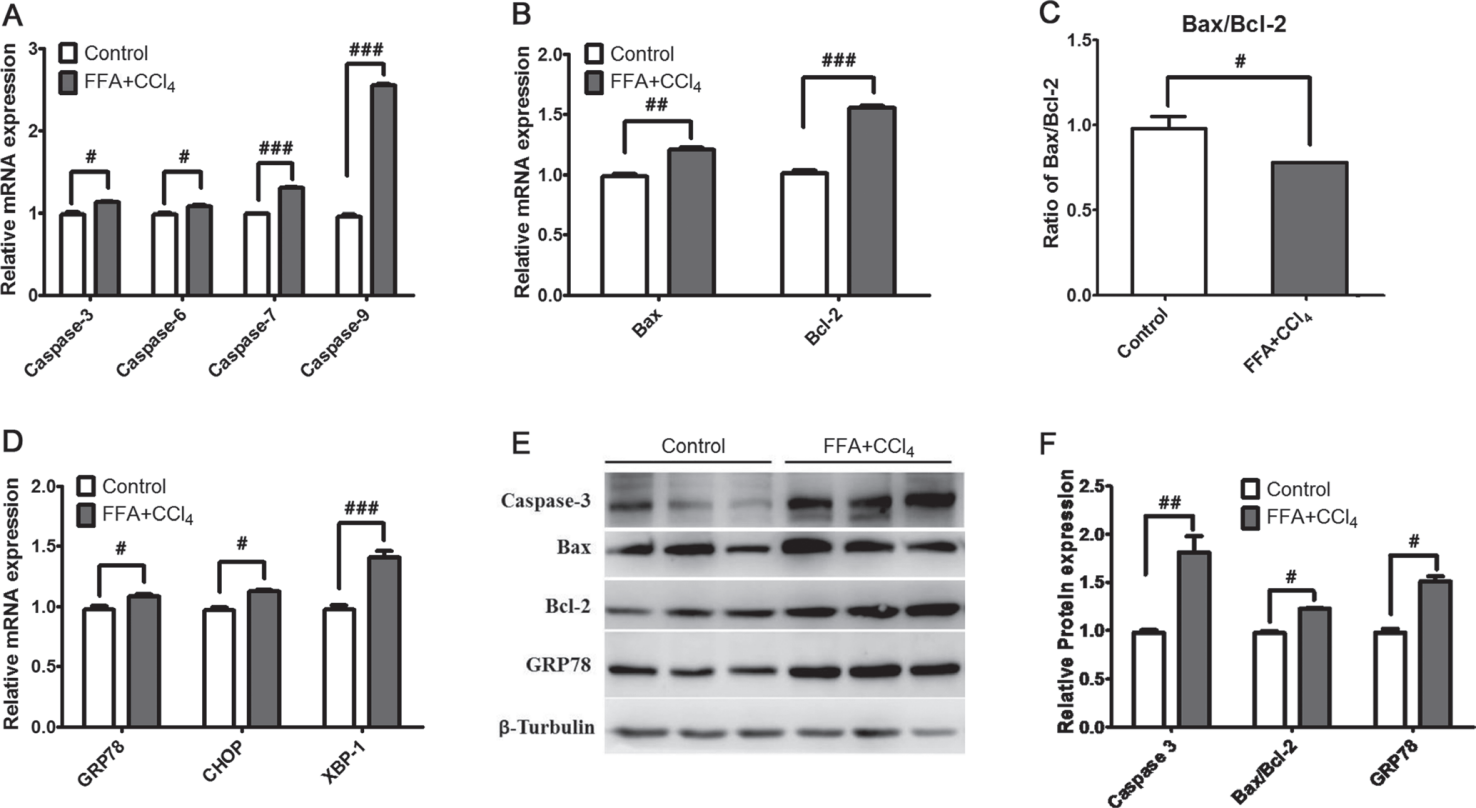
ER stress is associated with cell apoptosis The mRNA expression of (**A**) Caspase family genes (caspase-3, caspase-6, caspase-7 and caspase-9), (**B**) Bcl-2 family genes (Bax, Bcl-2), (**C**) the ratios of Bax/Bcl-2, and (**D**) molecular chaperones of ER (GRP78, CHOP, and XBP-1) were analysed by qRT-PCR, and the protein expression of caspase-3, Bax/Bcl-2, and GRP78 were determined by western blot (**E** and **F**). Data were presented as the mean ± sd of three independent experiments. ^#^*p* < 0.05, ^##^*p* < 0.01, ^###^*p* < 0.001 *vs* control cells.

The caspase family is a group of cysteine proteases with similar structure, and many members of this family are associated with apoptosis [28]. In FFA+CCl_4_ (4mM) stressed cells, the mRNA expression of caspase-3, caspase-6, caspase-7 and caspase-9 was all significantly increased (Figure 2A) and the caspase -3 protein expression was also increased (Figure 2D and 2E). The ratio of Bax/Bcl-2 is often used to evaluate apoptosis, while Bcl-2 inhibits apoptosis, the Bcl-2-associated X protein (Bax) promotes the process [29]. In our FFA+CCl_4_ stressed cells, both Bax and Bcl-2 mRNA (Figure 2B and 2C) and protein expression (Figure 2D and 2E) were significantly increased in comparison with control cells, however, the ratio of Bax/Bcl-2 was decreased (Figure 2C and 2E) indicating apoptotic status of FFA+CCl_4_ stressed cells.

ER stress is one of the major mechanisms associated with apoptosis [30, 31]. In FFA+CCl_4_ stressed cells, the expression of ER stress related molecules including GRP78 (Bip), CHOP, and XBP-1 all significant increased compared with control cells (Figure 2D). We further analysed the protein expression of GRP78, and found that the protein expression was consistent with the mRNA expression (Figure 2E and 2F), suggesting that hepatocyte apoptosis was related to ER stress.

### RSV inhibited apoptosis in stressed HepG2 cells

**Figure 3:**
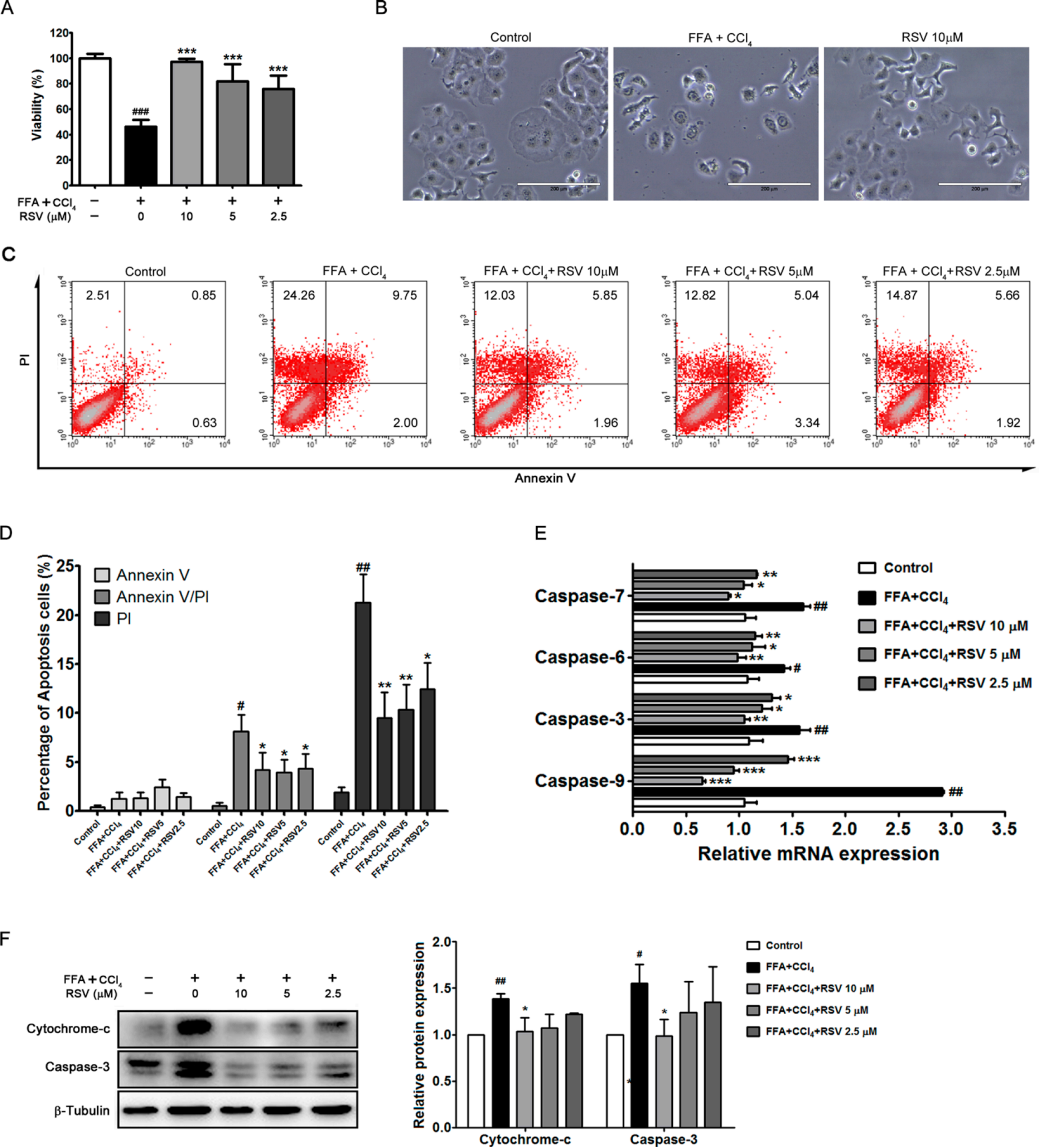
The effect of RSV on apoptotic cells HepG2 cells were first stressed by FFA and CCl_4_ (4 mM) solution, and then treated with RSV (2.5 μM, 5 μM and 10 μM) for 24 h, and cell viability was determined (**A**). Representative images of the morphological change upon 10μM RSV treatment were obtained (**B**), magnification, 200×). The effect of RSV treatment (2.5 μM, 5 μM and 10 μM) on apoptosis was indicated by annexin V (FITC)/PI binding assay (**C**), and the percentage of apoptotic cells was calculated (**D**). qRT-PCR analysis showed the genetic changes in response to RSV treatment (**E**), and western blot analysis showed the protein expression of cytochrome-c and caspase-3 of the cells (**F**). The data were presented as the mean ± sd of three independent experiments. ^#^*p* < 0.05, ^##^*p* < 0.01 *vs* control cells; *p < 0.05, ^*^p < 0.01, ^**^*p < 0.001 *vs* FFA+CCl_4_ stressed cells.

To evaluate the effect of RSV on apopotosis, we added different concentrations of RSV (2.5 μM, 5 μM, 10 μM) along with CCl_4_ solution (4 mM) to FFA stressed cells. Results showed that RSV could increase cell viability in a dose-dependent manner (Figure 3A). Cell morphology observation indicated that apoptotic features (cell shrinkage, nuclear fragmentation and chromatin condensation) were obviously improved in 10 μM RSV treated cells (Figure 3B). Based on flow cytometry analysis, RSV treatment decreased the percentage of cells that stained positive with Annexin V and/or PI in a dose-dependent manner (Figure 3C and 3D). Furthermore, RSV could significantly reduce the mRNA expression of caspase family members (caspase -3, -6, -7, -9) in a dose-dependent manner (Figure 3E). To further verified the effect, we detected the protein expression of caspase-3 and cytochrome c, whereas the expression significantly increased in FFA+CCl_4_ stressed cells, RSV treatment could restore the protein expression to nearly normal level (Figure 3F).

### RSV alleviates ER stress in stressed HepG2 cells

**Figure 4:**
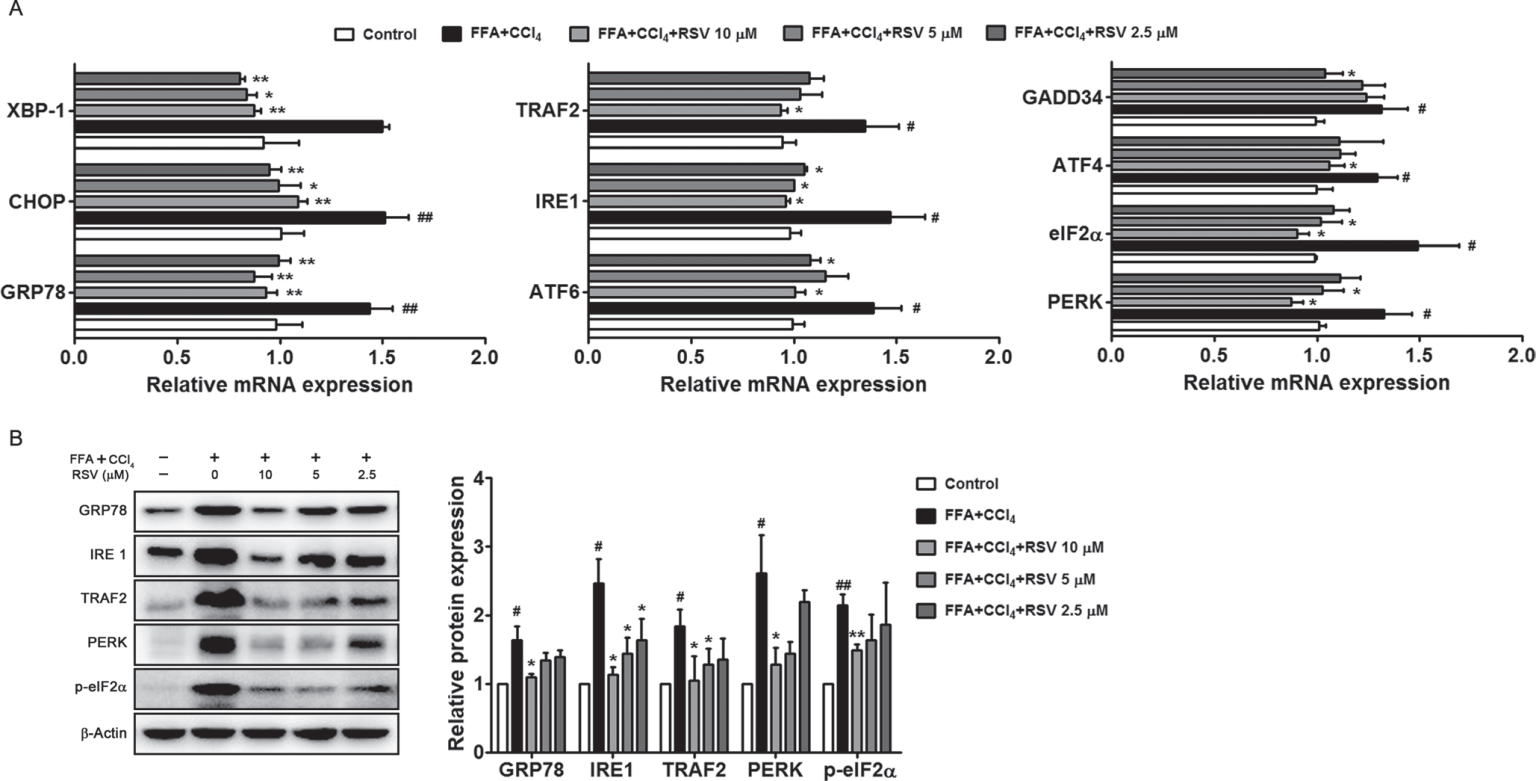
The effect of RSV on ER stress related molecules (**A**) The mRNA expression of ER stress-related genes (GRP78, CHOP, XBP-1, IRE1, TRAF2, PERK, ATF6, GADD34, eIF2α and ATF4) was analysed by qRT-PCR. (**B**) Western blot analysis of the GRP78, IRE1, TRAF2, PERK protein expression and eIF2α phosphorylation. The values are presented as the mean ± sd of three independent experiments. ^#^*p* < 0.05, ^##^*p* < 0.01 *vs* control cells; *p < 0.05 and ^*^p < 0.01 *vs* FFA+CCl_4_stressed cells.

Lipids or toxins accumulation could increase the susceptibility to ER stress, and ER stress results in inflammation and apoptosis via the activation of three different pathways: the inositol-requiring enzyme 1 (IRE1), RNA (PKR)-like ER kinase (PERK), and transcription factor 6 (ATF6) pathways [32–33]. Unexpectedly, we observed the increase of GRP78 expression in apoptotic cell, GRP78 as the essential protein to main cellular homeostasis via ca-ATPase, since CCl_4_ can direct act on mitochondria, the GRP78 increase of might result from stress response occurred in the early stage. To evaluate the effect of RSV on ER stress, we analysed the related molecules. Results showed that the expression of key molecules was significantly increased in FFA+CCl_4_ conditions, and RSV treatment could obviously decrease the related molecules, such as XBP-1, CHOP, TRAF2, IRE1, ATF6, GADD34, ATF4, eIF2α, PERK (Figure 4A). We further analysed the protein expression of several molecules, and found that RSV could inhibit GRP78, IRE1, TRAF2, PERK protein expression and eIF2α phosphorylation (Figure 4B). These results suggested that the RSV-induced inhibition of the ER stress response is one possible mechanisms of the anti-apoptotic effect.

### The effects of RSV were not completely dependent on GRP78

**Figure 5:**
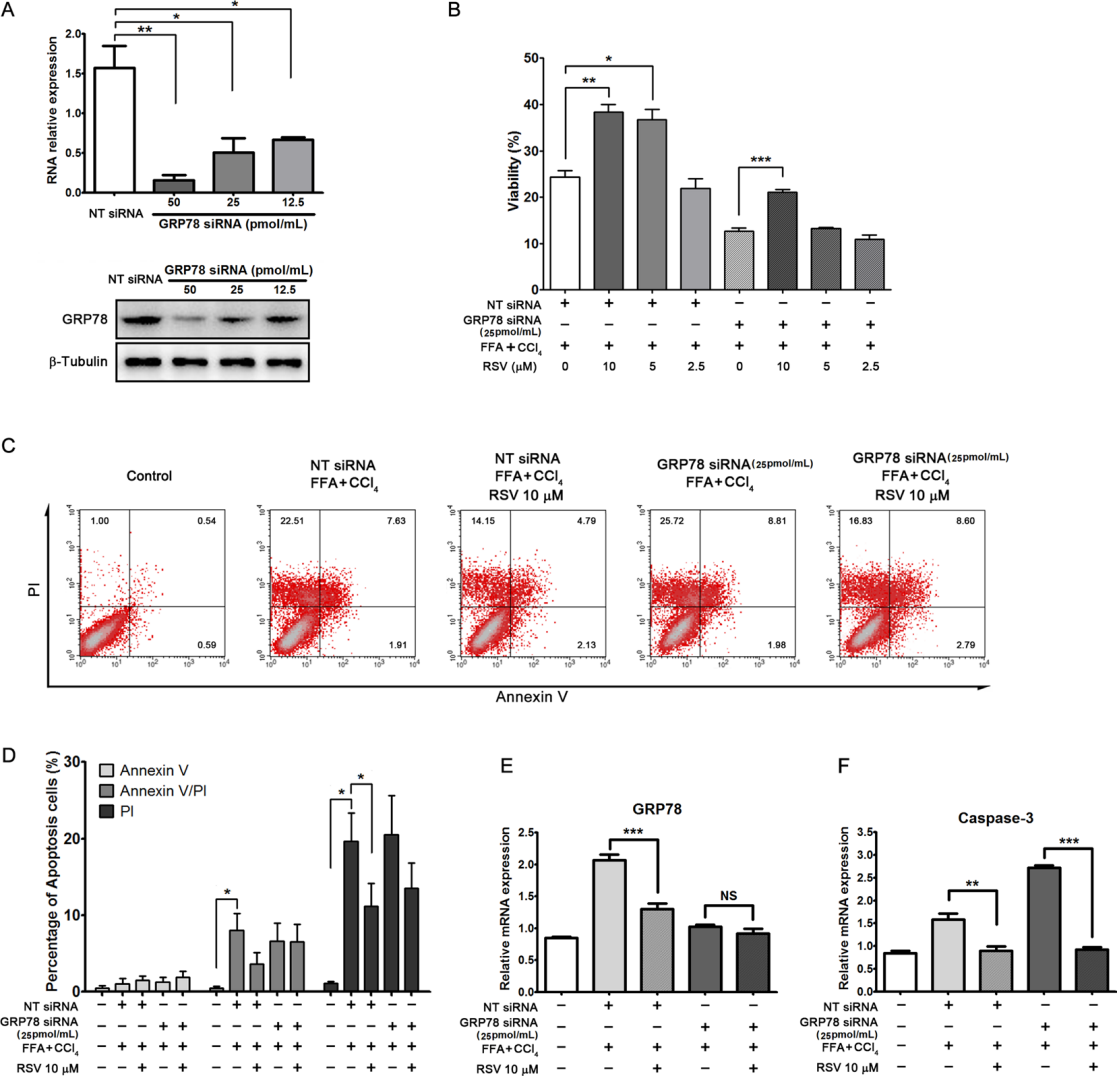
The effects of RSV on cells under GRP78 siRNA pre-treated condition (**A**) GRP78 silencing was measured by qRT-PCR and western blot. (**B**) The effects of RSV on cell viability in stressed HepG2 cells under GRP78 siRNA and non-targeting (NT) siRNA conditions. (**C**) Apoptosis analysis of the cells by the annexin V (FITC)/PI binding assay and the percentage of apoptotic cells was calculated (**D**). The mRNA experession of GRP78 (**E**) and caspase-3 (**F**) expression was determined by qRT-PCR. The data were presented as the mean ± sd of three independent experiments. NS= no significant difference, ^*^p < 0.01, ^*^p < 0.01,^**^*p < 0.001 *vs* the corresponding group.

GRP78 is a type of molecular chaperone that associates with IRE1, PERK, and ATF6 under stress-free conditions. When the stress response is initiated, GRP78 dissociate these proteins and activate the unfolded protein response (UPR) [34–35]. GRP78 knockout mice are susceptible to UPR activation and apoptosis, indicating that GRP78 is a negative regulator of the UPR and a potent suppressor of apoptosis [36, 37]. GRP78 small interfering RNA (siRNA) inhibited both the mRNA and protein expression of GRP78 in HepG2 cells (Figure 5A). To comprehensively evaluate RSV on apoptosis, 25 pmol/mL concentration of GRP78 siRNA was selected, and a non-targeting (high GC) siRNA (NT-siRNA) was used as a control. Cell viability was decrease in GRP78 siRNA cells, 10 μM RSV could restore the cell viability both in NT-siRNA cells and GRP78 siRNA cells (Figure 5B), and RSV also simultaneously reduced the percentage of apoptotic cells (Figure 5C and 5D). These results showed that RSV had a protective effect on apoptosis.

Previous results demonstrated that RSV down-regulated GRP78 expression in FFA+CCl_4_ stressed cells, but the effect was unobvious in GRP78 inhibited cells (Figure 5E). However, RSV decreased the expression of Caspase-3 both in NT-siRNA cells and GRP78 siRNA cells (Figure 5F). In GRP78 inhibited, FFA+CCl_4_ stressed cells, RSV could still inhibit apoptosis and increase viability, suggesting that the effect of RSV on apoptosis possibly occurs downstream of GRP78 in the ER stress pathway or via other pathways.

## DISCUSSION

Apoptosis plays an important role in liver injury. In the present study, we found that the lipid stressed HepG2 cells were susceptible to injury, and evaluated the anti-apoptotic effect of RSV. By analysing the ER stress related molecules, we indicated that the beneficial effects under RSV might be associated with ER function.

Cell apoptosis is programmed cell death and characterized by specific morphological features, such as cell shrinkage, nuclear fragmentation, and chromatin condensation. Apoptosis is an important mechanism in the progression and development of NAFLD, and hepatocyte apoptosis is usually observed in NAFLD patients and animal models [38, 39].

Apoptosis can be induced by the activation of death receptors, mitochondrial dysfunction, DNA damage, excessive autophagy, and ER stress [40]. In liver cells, mitochondrial dysfunction can amplify the apoptotic signal and integrate various pathways to promote apoptotic response. The caspase family and the Bcl-2 family play important roles in this process. Mitochondrial dysfunction causes the release of cytochrome c into the cytosol. Subsequently, cytochrome c can combine with caspase-9 and apoptotic-protein activation factor-1 (Apaf-1) to start the apoptosis programme by forming the apoptosome, and the complex initiates further cascades that leading to apoptosis [41]. In addition, Bcl-2 family proteins regulate mitochondrial function, and the Bax/Bcl-2 ratio usually indicates the extent of apoptosis [42]. In the present study, the levels of caspase-3, caspase-6, caspase-7 and caspase-9 were significantly increased in the stressed cells, which confirmed the pathological mechanism underlying the apoptosis. The Bax/Bcl-2 ratio was significantly decreased though the expression of pro-apoptosis Bax and the anti-apoptotic Bcl-2 was increased. In addition, the anti-apoptosis function of Bcl-2 might be associated with the adaptive mechanisms of negative feedback [43].

ER stress plays a special role in NAFLD, to cope with ER stress, cells activate UPR. When the UPR is too strong or too long, the stress response cannot restore ER homeostasis, and signals downstream of the ER stress will activate the apoptotic process. RSV is believed to have liver-protective and anti-inflammatory effects. Previous research indicated that RSV could alleviate the onset of NAFLD in a mouse model and inhibit the ER stress during the progression of the disease [27]. Consistently, we showed that RSV could inhibit apoptosis in stressed HepG2 cells and the transcription of the corresponding genes, suggesting that RSV had a protective effect on liver cells under apoptosis-inducing conditions. Other study revealed that resveratrol ameliorates ER stress by down-regulating CHOP and GRP78 expression and hampering caspase-3 activity, which is also agreed to our data [44]. In addition, RSV could decrease the transcription of ER stress related molecules, having an overall inhibitory effect on the ER stress. GRP78 is molecular chaperone located in the ER, although the expression of GRP78 increased during apoptosis, GRP78 knock out animals are prone to apoptosis [45]. However, when the GRP78 gene was down-regulated with siRNA, RSV still played a role in inhibiting apoptosis in the model cells, indicating that the anti-apoptotic effect of RSV was not solely dependent on GRP78 and has an overall inhibitory effect on the ER stress reaction. Actually, studies showed that RSV could protect cells from apoptosis by directly inhibit Caspase-3 [46], thus the anti-apoptotic effect of RSV might be dependent on both caspase inhibition and ER stress restoration.

In summary, the present study demonstrated that lipid stressed HepG2 cells were susceptible to injury, and the natural compound RSV could improve apoptosis in lipid and injury stressed cells, one of the possible mechanisms is ER function restoration.

## MATERIALS AND METHODS

### Cell culture

HepG2 cells (Shanghai Genetics Chinese Academy of Sciences Cell Bank, Shanghai, China), a cell line derived from a polarized human liver, were maintained in Dulbecco's modified Eagle's medium (DMEM, high glucose) supplemented with 1% (v/v) antibiotic-antimycotic solution (Gibco, U.S.A.) and 10% (v/v) heat-inactivated foetal bovine serum (Gibco, U.S.A.). The cells were incubated at 37°C with 95% humidity and 5% CO_2_.

An FFA solution was prepared by mixing oleic acid and palmitic acid (2:1) and dissolving the mixture in DMEM medium under appropriate heating conditions. The toxin solution contained CCl_4_ and DMSO, which were suspended in DMEM medium via ultrasonic dispersion.

HepG2 cells were seeded at a density of 5 × 10^3^ cells/well in 96-well plates or 1.25 × 10^5^ cells/well in 6-well plates and incubated overnight. Then, the cells were co-cultured with FFA solution (final concentrations of 0.5 mM oleic acid and 0.25 mM palmitic acid) for 24 h. For apoptotic induction, the cells were then exposed to the CCl_4_ (1 mM, 2 mM, 4 mM, 6 mM) dissolved in 0.15% DMSO for another 24 h. RSV at different concentrations (2.5 μM, 5 μM, 10 μM) was added simultaneously with CCl_4_ solution (4 mM) to evaluate the therapeutic effect.

### Oil red O staining and Nile red staining

After 24 h co-culture with FFA solution, HepG2 cells were washed two times with phosphate-buffered saline (PBS). Cells were stained with Oil Red O staining solution for 10 min at 18–20°C and counterstained with haematoxylin for 20 s. The stained cells were photographed under a microscope (EVOS, AMG, U.S.A.).

For Nile Red staining, the cells were digested with trypsin-EDTA and collected in a tube by centrifugation. The cells were stained with Nile red at 4°C for 20 min. The stained cells were detected using flow cytometry (FACSCalibur, BD Biosciences, U.S.A.). Data analysis was performed using CellQuest software.

### Cell apoptosis assay

After treatment, cells were washed two times with PBS and stained with annexin V (FITC) in binding buffer at 4°C for 20 min. The cells were then suspended in binding buffer and stained with propidium iodide (PI). Cell fluorescence was detected by flow cytometry (FACSCalibur, BD Biosciences, U.S.A.). CellQuest software was used to analyse the data.

### Cell viability

The crystal violet assay was used to detect cell viability. At the end of each experiment, crystal violet solution (0.03% crystal violet dissolved in 20% methanol) was used to fix and stain the cells for 10 min, and then lysis buffer was used to solubilize the dyed samples. After the dye was completely dissolved, the absorbance values were read at 540/630 nm using a spectrometer. Cell viability was calculated based on the absorbance value.

### Quantitative real-time PCR analysis

At the end of the each experiment, HepG2 cells were lysed with TRIzol reagent (Invitrogen, U.S.A.), and RNA was extracted for reverse transcription using a PrimeScript™ RT Reagent Kit (Takara, Japan). Real-time PCR was performed using SYBR Green Reagents (Takara, Japan) with quantitative analysis using a Real-Time PCR System (Applied Biosystems, U.S.A.). The primer sequences for the human samples were listed in Table 1. The amplification reactions, data acquisition and analyses were performed using 7500 Software v2.0.6 (Applied Biosystems, U.S.A.), and the relative mRNA expression was calculated using the 2^-ΔΔCT^ method.

**Table 1:** List of primer sequences for real-time PCR analysis

Gene	Primer sequence (5′ to 3′)	NCBI GeneID
*caspase-3*	GAAATTGTGGAATTGATGCGTGACTACAACGATCCCCTCTGAAAAA	638
*caspase-6*	CACCAACATAACTGAGGTGGATGAGGAGGAGCCATATTTTCCCA	839
*caspase-7*	AGTGACAGGTATGGGCGTTCCGGCATTTGTATGGTCCTCTT	840
*caspase-9*	CTGTCTACGGCACAGATGGATGGGACTCGTCTTCAGGGGAA	842
*grp78*	CATCACGCCGTCCTATGTCGCGTCAAAGACCGTGTTCTCG	3309
*chop*	CTCTGATTGACCGAATGGTGAAGGGACTGATGCTCCCAATTG	1649
*xbp1*	CCCTCCAGAACATCTCCCCATACATGACTGGGTCCAAGTTGT	7494
*bax*	CCCGAGAGGTCTTTTTCCGAGCCAGCCCATGATGGTTCTGAT	581
*bcl2*	GGTGGGGTCATGTGTGTGGCGGTTCAGGTACTCAGTCATCC	596
*ire1*	CACAGTGACGCTTCCTGAAACGCCATCATTAGGATCTGGGAGA	2081
*traf2*	GCTCATGCTGACCGAATGTCGCCGTCACAAGTTAAGGGGAA	7186
*perk*	GGAAACGAGAGCCGGATTTATTACTATGTCCATTATGGCAGCTTC	9451
*atf6*	TCCTCGGTCAGTGGACTCTTACTTGGGCTGAATTGAAGGTTTTG	22926
*gadd34*	ATGATGGCATGTATGGTGAGCAACCTTGCAGTGTCCTTATCAG	23645
*eif2α*	TGGTGAATGTCAGATCCATTGCTAGAACGGATACGCCTTCTGG	1965
*atf4*	ATGACCGAAATGAGCTTCCTGGCTGGAGAACCCATGAGGT	478
*β-actin*	CATGTACGTTGCTATCCAGGCCTCCTTAATGTCACGCACGAT	60

### Western blotting analysis

At the end of the each experiment, HepG2 cells were placed in RIPA lysis buffer (Beyotime Biotechnology, China) and centrifuged (12,000 × g for 5 min) to remove impurities. Proteins were quantitated using the bicinchoninic acid (BCA) method. Protein samples (total protein, 50 μg/lane) were examined using 10% polyacrylamide gel electrophoresis and then transferred to membranes (polyvinylidene fluoride, PVDF, Millipore, U.S.A.). Non-specific binding was blocked with 5% bovine serum albumin (BSA) with 0.05% Tween in triethanolamine-buffered saline solution (TBS) and incubated with primary antibodies (cytochrome c, caspase-3, GRP78, phospho-eIF-2α, IRE1, TRAF2, PERK, β-tubulin and β-actin; all antibodies were acquired from Cell Signaling Technology and Abcam, U.S.A.) at 4°C for 12 h, followed by the addition of HRP-conjugated secondary antibodies (ICLLAB, U.S.A.). The membranes were washed three times with TBST (TBS containing 0.05% Tween). Bands were visualized and quantified by gel imaging instrument and matched software (ImageQuant LAS system, GE Healthcare Life Sciences, U.S.A.).

### GRP78 gene silencing

GRP78 gene silencing was achieved by the transfection of a specific small interfering (si) (5′-CAG CAA CTG GTT AAA GAG TTC A-3′) RNA targeting GRP78 mRNA (Stealth siRNA™, Invitrogen, U.S.A.), and the negative control cells were added with a non-targeting (NT) siRNA (Stealth siRNA™ Negative Control, high GC, Invitrogen, U.S.A.). The cells were transfected with Lipofectamine RNAiMAX reagent for 24 h using siRNAs at final concentrations of 12.5, 25, and 50 nM. The transfection efficiency was evaluated using qPCR, and the down-regulation of GRP78 was measured by Western blotting.

### Statistical analyses

All the data was analyzed using GraphPad Prism 5.01 software (GraphPad Prism Software Inc., San Diego, CA) and presented as Mean ± standard deviation (SD). Student's *t* test was used when the data had a normal distribution. *p* < 0.05 was considered statistically significant.

## Abbreviations

NAFLD: non-alcoholic fatty liver disease
NASH: non-alcoholic steatohepatitis
ER: endoplasmic reticulum
RSV: resveratrol
FFA: free fatty acid
MFI: mean fluorescence intensity
CCl_4_: carbon tetrachloride
MMP: mitochondrial membrane potential
Bax: Bcl-2-associated X protein
IRE1: inositol-requiring enzyme 1
PERK: RNA (PKR)-like ER kinase
ATF6: transcription factor 6
UPR: unfolded protein response
siRNA: small interfering RNA
Apaf-1: apoptotic-protein activation factor-1
DMEM: Dulbecco's modified Eagle's medium
PBS: phosphate-buffered saline
PI: propidium iodide
BCA: bicinchoninic acid
TBS: triethanolamine-buffered saline solution
SD: standard deviation

## References

[1] Trefts E, Williams AS, Wasserman DH (2015). Exercise and the Regulation of Hepatic Metabolism. Prog Mol Biol Transl Sci.

[2] Ress C, Kaser S (2016). Mechanisms of intrahepatic triglyceride accumulation. World J Gastroenterol.

[3] Onyekwere CA, Ogbera AO, Samaila AA, Balogun BO, Abdulkareem FB (2015). Nonalcoholic fatty liver disease: Synopsis of current developments. Niger J Clin Pract.

[4] Tanaka K, Masaki Y, Tanaka M, Miyazaki M, Enjoji M, Nakamuta M, Kato M, Nomura M, Inoguchi T, Kotoh K, Takayanagi R (2014). Exenatide improves hepatic steatosis by enhancing lipid use in adipose tissue in nondiabetic rats. World J Gastroenterol.

[5] Wei Y, Wang D, Pagliassotti MJ (2007). Saturated fatty acid-mediated endoplasmic reticulum stress and apoptosis are augmented by trans-10, cis-12-conjugated linoleic acid in liver cells. Mol Cell Biochem.

[6] Pfaffenbach KT, Gentile CL, Nivala AM, Wang D, Wei Y, Pagliassotti MJ (2010). Linking endoplasmic reticulum stress to cell death in hepatocytes: roles of C/EBP homologous protein and chemical chaperones in palmitate-mediated cell death. Am J Physiol Endocrinol Metab.

[7] Xu C, Bailly-Maitre B, Reed JC (2005). Endoplasmic reticulum stress: cell life and death decisions. J Clin Invest.

[8] Zhang K, Kaufman RJ (2006). The unfolded protein response: a stress signaling pathway critical for health and disease. Neurology.

[9] Baur JA, Pearson KJ, Price NL, Jamieson HA, Lerin C, Kalra A, Prabhu VV, Allard JS, Lopez-Lluch G, Lewis K, Pistell PJ, Poosala S, Becker KG (2006). Resveratrol improves health and survival of mice on a high-calorie diet. Nature.

[10] Baur JA, Sinclair DA (2006). Therapeutic potential of resveratrol: the in vivo evidence. Nat Rev Drug Discov.

[11] Hung LM, Chen JK, Huang SS, Lee RS, Su MJ (2000). Cardioprotective effect of resveratrol, a natural antioxidant derived from grapes. Cardiovasc Res.

[12] Das S, Das DK (2007). Resveratrol: a therapeutic promise for cardiovascular diseases. Recent Pat Cardiovasc Drug Discov.

[13] Delmas D, Lancon A, Colin D, Jannin B, Latruffe N (2006). Resveratrol as a chemopreventive agent: a promising molecule for fighting cancer. Curr Drug Targets.

[14] Das S, Das DK (2007). Anti-inflammatory responses of resveratrol. Inflamm Allergy Drug Targets.

[15] de la Lastra CA, Villegas I (2007). Resveratrol as an antioxidant and pro-oxidant agent: mechanisms and clinical implications. Biochem Soc Trans.

[16] Bujanda L, Hijona E, Larzabal M, Beraza M, Aldazabal P, Garcia-Urkia N, Sarasqueta C, Cosme A, Irastorza B, Gonzalez A, Arenas JI (2008). Resveratrol inhibits nonalcoholic fatty liver disease in rats. BMC Gastroenterol.

[17] Shang J, Chen LL, Xiao FX, Sun H, Ding HC, Xiao H (2008). Resveratrol improves non-alcoholic fatty liver disease by activating AMP-activated protein kinase. Acta Pharmacol Sin.

[18] Vetterli L, Maechler P (2011). Resveratrol-activated SIRT1 in liver and pancreatic beta-cells: a Janus head looking to the same direction of metabolic homeostasis. Aging (Albany NY).

[19] Andrade JM, Paraiso AF, de Oliveira MV, Martins AM, Neto JF, Guimaraes AL, de Paula AM, Qureshi M, Santos SH (2014). Resveratrol attenuates hepatic steatosis in high-fat fed mice by decreasing lipogenesis and inflammation. Nutrition.

[20] Zhu W, Chen S, Li Z, Zhao X, Li W, Sun Y, Zhang Z, Ling W, Feng X (2014). Effects and mechanisms of resveratrol on the amelioration of oxidative stress and hepatic steatosis in KKAy mice. Nutr Metab (Lond).

[21] Graham RM, Hernandez F, Puerta N, De Angulo G, Webster KA, Vanni S (2016). Resveratrol augments ER stress and the cytotoxic effects of glycolytic inhibition in neuroblastoma by downregulating Akt in a mechanism independent of SIRT1. Exp Mol Med.

[22] Gu S, Chen C, Jiang X, Zhang Z (2016). ROS-mediated endoplasmic reticulum stress and mitochondrial dysfunction underlie apoptosis induced by resveratrol and arsenic trioxide in A549 cells. Chem Biol Interact.

[23] Gwak H, Kim S, Dhanasekaran DN, Song YS (2016). Resveratrol triggers ER stress-mediated apoptosis by disrupting N-linked glycosylation of proteins in ovarian cancer cells. Cancer Lett.

[24] Lin Y, Zhu J, Zhang X, Wang J, Xiao W, Li B, Jin L, Lian J, Zhou L, Liu J (2016). Inhibition of Cardiomyocytes Hypertrophy by Resveratrol Is Associated with Amelioration of Endoplasmic Reticulum Stress. Cell Physiol Biochem.

[25] Li A, Zhang S, Li J, Liu K, Huang F, Liu B (2016). Metformin and resveratrol inhibit Drp1-mediated mitochondrial fission and prevent ER stress-associated NLRP3 inflammasome activation in the adipose tissue of diabetic mice. Mol Cell Endocrinol.

[26] Tabata Y, Takano K, Ito T, Iinuma M, Yoshimoto T, Miura H, Kitao Y, Ogawa S, Hori O, Vaticanol B (2007). a resveratrol tetramer, regulates endoplasmic reticulum stress and inflammation. Am J Physiol Cell Physiol.

[27] Pan QR, Ren YL, Liu WX, Hu YJ, Zheng JS, Xu Y, Wang G (2015). Resveratrol prevents hepatic steatosis and endoplasmic reticulum stress and regulates the expression of genes involved in lipid metabolism, insulin resistance, and inflammation in rats. Nutr Res.

[28] Wu H, Che X, Zheng Q, Wu A, Pan K, Shao A, Wu Q, Zhang J, Hong Y (2014). Caspases: a molecular switch node in the crosstalk between autophagy and apoptosis. Int J Biol Sci.

[29] Zhou F, Yang Y, Xing D (2011). Bcl-2 and Bcl-xL play important roles in the crosstalk between autophagy and apoptosis. FEBS J.

[30] Ozcan U, Yilmaz E, Ozcan L, Furuhashi M, Vaillancourt E, Smith RO, Gorgun CZ, Hotamisligil GS (2006). Chemical chaperones reduce ER stress and restore glucose homeostasis in a mouse model of type 2 diabetes. Science.

[31] Zhang XQ, Pan Y, Yu CH, Xu CF, Xu L, Li YM, Chen WX (2015). PDIA3 Knockdown Exacerbates Free Fatty Acid-Induced Hepatocyte Steatosis and Apoptosis. PLoS One.

[32] Wang D, Wei Y, Pagliassotti MJ (2006). Saturated fatty acids promote endoplasmic reticulum stress and liver injury in rats with hepatic steatosis. Endocrinology.

[33] Ron D, Walter P (2007). Signal integration in the endoplasmic reticulum unfolded protein response. Nat Rev Mol Cell Biol.

[34] Zhu G, Lee AS (2015). Role of the unfolded protein response, GRP78 and GRP94 in organ homeostasis. J Cell Physiol.

[35] Schroder M, Kaufman RJ (2005). The mammalian unfolded protein response. Annu Rev Biochem.

[36] Pyrko P, Schonthal AH, Hofman FM, Chen TC, Lee AS (2007). The unfolded protein response regulator GRP78/BiP as a novel target for increasing chemosensitivity in malignant gliomas. Cancer Res.

[37] Reddy RK, Mao C, Baumeister P, Austin RC, Kaufman RJ, Lee AS (2003). Endoplasmic reticulum chaperone protein GRP78 protects cells from apoptosis induced by topoisomerase inhibitors: role of ATP binding site in suppression of caspase-7 activation. J Biol Chem.

[38] Xu Z, Zhang X, Lau J, Yu J (2016). C-X-C motif chemokine 10 in non-alcoholic steatohepatitis: role as a pro-inflammatory factor and clinical implication. Expert Rev Mol Med.

[39] Tanaka S, Hikita H, Tatsumi T, Sakamori R, Nozaki Y, Sakane S, Shiode Y, Nakabori T, Saito Y, Hiramatsu N, Tabata K, Kawabata T, Hamasaki M (2016). Rubicon inhibits autophagy and accelerates hepatocyte apoptosis and lipid accumulation in nonalcoholic fatty liver disease in mice. Hepatology.

[40] Fernandez A, Ordonez R, Reiter RJ, Gonzalez-Gallego J, Mauriz JL (2015). Melatonin and endoplasmic reticulum stress: relation to autophagy and apoptosis. J Pineal Res.

[41] Linder M, Tschernig T (2016). Vasculogenic mimicry: Possible role of effector caspase-3, caspase-6 and caspase-7. Ann Anat.

[42] Shukla S, Rizvi F, Raisuddin S, Kakkar P (2014). FoxO proteins’ nuclear retention and BH3-only protein Bim induction evoke mitochondrial dysfunction-mediated apoptosis in berberine-treated HepG2 cells. Free Radic Biol Med.

[43] Wu H, Qiu Y, Shu Z, Zhang X, Li R, Liu S, Chen L, Liu H, Chen N (2016). Protective effect of Trillium tschonoskii saponin on CCl4-induced acute liver injury of rats through apoptosis inhibition. Can J Physiol Pharmacol.

[44] Gaballah HH, Zakaria SS, Elbatsh MM, Tahoon NM (2016). Modulatory effects of resveratrol on endoplasmic reticulum stress-associated apoptosis and oxido-inflammatory markers in a rat model of rotenone-induced Parkinson's disease. Chem Biol Interact.

[45] Luo S, Mao C, Lee B, Lee AS (2006). GRP78/BiP is required for cell proliferation and protecting the inner cell mass from apoptosis during early mouse embryonic development. Mol Cell Biol.

[46] Ulakcsai Z, Bagamery F, Vincze I, Szoko E, Tabi T (2015). Protective effect of resveratrol against caspase 3 activation in primary mouse fibroblasts. Croat Med J.

